# Recent developments in the understanding of the interactions between the vestibular system, memory, the hippocampus, and the striatum

**DOI:** 10.3389/fneur.2022.986302

**Published:** 2022-09-02

**Authors:** Paul F. Smith

**Affiliations:** ^1^Department of Pharmacology and Toxicology, School of Biomedical Sciences and Brain Health Research Centre, University of Otago, Dunedin, New Zealand; ^2^Eisdell Moore Centre for Hearing and Balance Research, University of Auckland, Auckland, New Zealand

**Keywords:** vestibular system, memory, hippocampus, Alzheimer's disease, striatum, Parkinson's disease

## Abstract

Over the last two decades, evidence has accumulated to demonstrate that the vestibular system has extensive connections with areas of the brain related to spatial memory, such as the hippocampus, and also that it has significant interactions with areas associated with voluntary motor control, such as the striatum in the basal ganglia. In fact, these functions are far from separate and it is believed that interactions between the striatum and hippocampus are important for memory processing. The data relating to vestibular-hippocampal-striatal interactions have considerable implications for the understanding and treatment of Alzheimer's Disease and Parkinson's Disease, in addition to other neurological disorders. However, evidence is accumulating rapidly, and it is difficult to keep up with the latest developments in these and related areas. The aim of this review is to summarize and critically evaluate the relevant evidence that has been published over the last 2 years (i.e., since 2021), in order to identify emerging themes in this research area.

## Introduction

The last two decades have seen significant advances in our understanding of the contributions of the vestibular system to functions beyond the vestibulo-ocular and vestibulo-spinal reflexes, such as higher cognitive functions [see (1) for a recent review]. This evidence has obvious implications for the etiology and treatment of dementia, such as Alzheimer's Disease (AD) [see (2) for a review]. To a lesser extent, evidence has been gradually accumulating to suggest that the vestibular system has a significant impact on voluntary motor control, though connections with the striatum, a part of the basal ganglia that is also important for memory [see (3) for a review]. This evidence has important implications for our understanding and treatment of Parkinson's Disease (PD) as well as other disorders of the basal ganglia. In the last 2 years, many studies in these two areas have been published, consolidating existing hypotheses and advancing new ones. The aim of this review is to summarize and critically evaluate what I consider to be the most important of these studies, with a view to delineating some of the next steps in these research areas. Since this Special Topic concerns “Insights in Neuro-Otology: 2021”, papers published prior to 2021 will not be included, unless they are directly relevant to the topic under discussion, and no attempt will be made to be exhaustive.

## The vestibular system and cognitive function

### Spatial memory in humans

Although studies of the relationship between the vestibular system and cognition date back to the 1960's and 1970's, by the 2000's, systematic, well-controlled behavioral studies were reported which demonstrated the importance of the vestibular system for spatial memory, in particular [see (1) for a recent review]. These studies were initially mostly animal studies, but since then many studies have been published that support the hypothesis that vestibular loss can cause varying degrees of cognitive impairment in patients with vestibular disorders (1). At the same time, animal studies have revealed that vestibular loss is associated with the dysfunction of various spatially-responsive neurons in the brain, such thalamic head direction cells, hippocampal place cells and entorhinal grid cells (1). Even in just the last 2 years, many studies have been published which support and extend the view that the vestibular system makes important contributions to memory, especially spatial memory.

Xie et al. (4) studied cognitive function in 126 neuro-otology clinic outpatients, using interviews and cognitive questionnaire scores. Sixty percent of the patients reported experiencing cognitive problems. Using linear regression, they found that the patients, compared to non-vertiginous controls, scored significantly worse on the total Neuropsychological Vertigo Inventory (NVI, which measures cognitive, emotional, visual, and motor symptoms), and the NVI cognitive composite and 3 individual NVI cognition subscales (i.e., Attention, Space Perception, and Time Perception), but not the Everyday Memory Questionnaire (EMQ; tests general memory ability). The cognitive questionnaire scores were also positively correlated with the overall Depression, Anxiety and Stress Scale (DASS) scores, suggesting a relationship with emotional symptoms. The patients exhibited a heterogeneous range of vestibular disorders (e.g., vestibular migraine, Meniere's Disease, Benign Paroxsymal Positional Vertigo) and their hearing status was not clear. This is an important issue in terms of separating the contributions of vestibular vs. auditory deficits to cognitive dysfunction [see (5) for a discussion].

Schöberl et al. (6) studied 14 patients with either complete or incomplete bilateral vestibulopathy (BVP) and compared their performance with age-matched healthy controls in a navigation task requiring the retracing of familiar spatial routes and the combination of novel routes in order to locate objects in real space. [^18^F]-Fluorodeoxyglucose PET was used to describe their brain activation during the task and the subjects also wore a gaze-controlled, head fixed camera in order to quantify visual search behavior. Although the performance of the patients was not significantly different from the controls when retracing familiar routes, they performed significantly worse when having to combine novel routes, which was correlated with the degree of the BVP. At the same time, the right hippocampus and entorhinal cortex exhibited lower activity and the bilateral parahippocampal areas more activity in the patients during the navigation process. The results of this study suggest that vestibular loss may specifically affect the neural representation of *novel* spatial information.

Gammeri et al. (7) studied the navigation strategies of patients with vestibular loss using a virtual reality reverse T-maze to distinguish “allocentric” (a spatial strategy based on external landmarks) and “egocentric” (response, e.g., left vs. right turn) strategies. They compared 23 patients with unilateral vestibular loss (UVL) to 23 with bilateral vestibular loss (BVL) and 23 healthy controls matched for age, sex and education. The authors reported that for both the UVL and BVL groups, the odds of using a specific egocentric or allocentric strategy in the T maze were reduced. They observed that only a right UVL appeared to reduce the odds of adopting an allocentric spatial strategy. For those patients who used a specific strategy to navigate, the chances of using an allocentric strategy were reduced for the BVL patients, whereas the chances of using an egocentric strategy were reduced for the UVL patients. The authors acknowledged that hearing loss was a possible confounding factor in the study.

**Figure 1:**
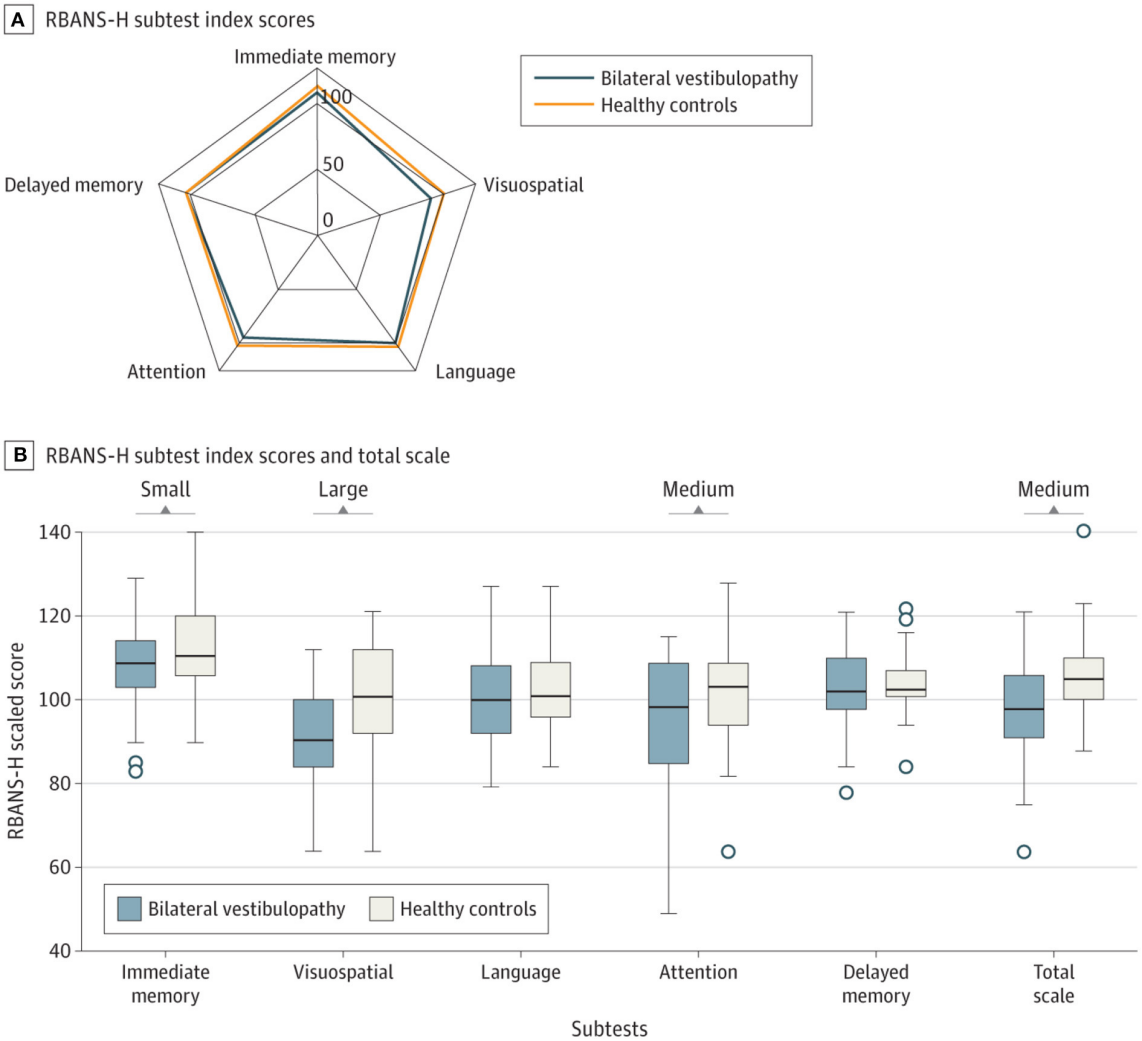
Effects of bilateral vestibulopathy on the Repeatable Battery for the Assessment of Neuropsychological Status (RBANS-H) scores in different cognitive domains. Comparison of RBANS-H scores between individuals with bilateral vestibulopathy and their matched healthy controls. **(A)** The relationship between the scores for immediate memory, delayed memory, attention, language and visuospatial function for bilateral vestibulopathy patients and healthy controls. **(B)** Whiskers indicate range; boxes, interquartile range (IQR); circles indicate outliers; bold line, median *d*. Small (Cohen's *d* = 0.2), medium (Cohen's *d* = 0.5), and large (Cohen's = 0.8) indicate clinically meaningful Cohen's *d* effect sizes. While there were medium and large differences for attention and visuospatial memory, there were only small effects for immediate memory, language and delayed memory. Reproduced from Bosmans et al. (8).

Bosmans et al. (8) studied cognitive function in 34 patients with bilateral vestibulopathy (BVP) compared to 34 controls, matched for age, sex and hearing status. BVP patients performed worse on the Repeatable Battery for the Assessment of Neuropsychological Status (RBANS-H) relative to the controls on all subscales, but with a medium statistical effect size (i.e., Cohen's *d*) for attention and a large effect size for visuospatial processing (i.e., processing visual stimuli in order to understand spatial relationships) (Figure 1). The authors found a positive correlation between Performance-Oriented Mobility Assessment (POMA) scores and RBANS-H scores, suggesting that the cognitive deficits were related to the degree of balance and gait dysfunction. An important aspect of this study was that the subjects were matched for hearing status, and therefore hearing loss cannot easily explain the cognitive deficits in the BVP patients (5).

Bigelow et al. (9) and Lacroix et al. (10) have recently published studies indicating that the vestibular system is important for a child's cognitive development. Stimulated by such studies, Van Hecke et al. (11) have published a new protocol to further investigate the involvement of the vestibular system in children's cognitive development. This will be an important area of investigation for developmental problems in children.

**Figure 2:**
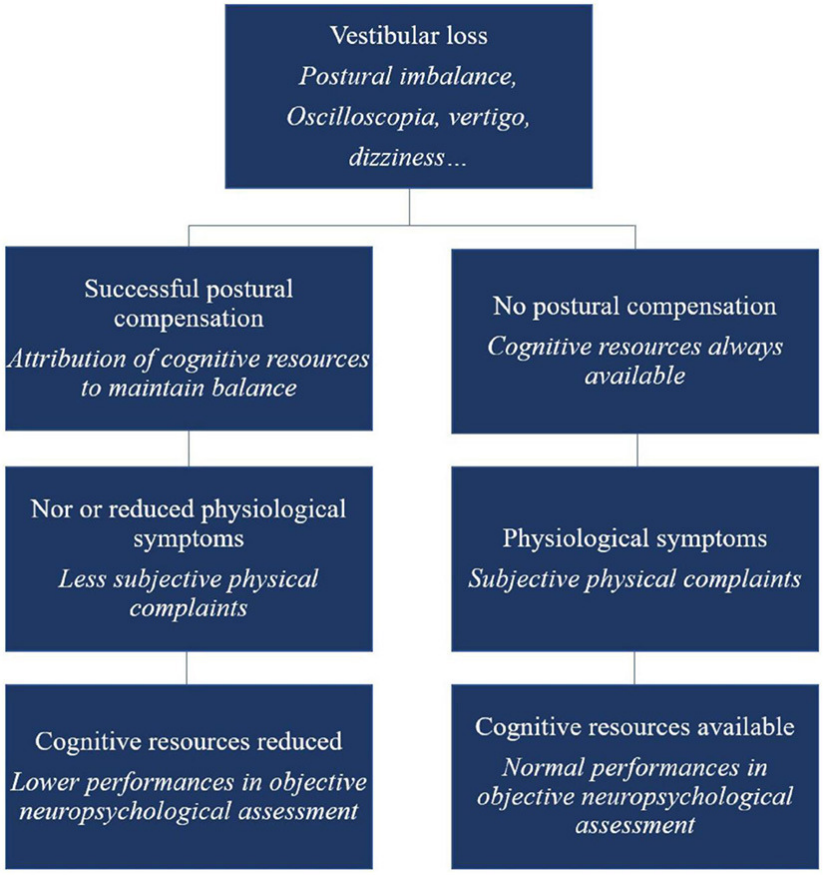
Schematic overview of the cognitive-vestibular compensation hypothesis—adaptation of the Kahneman's Capacity Model of Attention to vestibular damaged patients. Reproduced from Lacroix et al. (12).

Lacroix et al. (12) have suggested that Kahneman's Capacity Model of Attention might be usefully applied to the understanding of cognitive deficits associated with vestibular loss, and especially the variability in these amongst patients. The concept is that there is a limited quantity of cognitive resources that can be allocated to cognitive tasks during recovery from vestibular loss. They suggest that those patients who exhibit cognitive impairment may do so partly as a cost of compensating for their vestibular loss, whereas those people who not compensate very effectively, still have their full cognitive capacity (see Figure 2). This may be why the cognitive effects of vestibular dysfunction vary so much from one clinical study to another.

### Spatial orientation in humans

Borel et al. (13) studied the effects of unilateral vestibular neurotomy on the perception of the subjective straight ahead (SSA). They found that during the early phase of the recovery, the patients exhibited the typical translatory and rotatory bias toward the operated side; however, by 2 months post-op., they exhibited a translatory rightward bias of the SSA, without a rotation bias, irrespective of the side of the neurotomy. The authors interpreted these findings as indicative of a long-term change in body representation which is reminiscent of spatial neglect following hemispheric lesions. Both the left and right neurotomy groups suffered hearing loss of approximately equal magnitude.

### Brain volume and Alzheimer's disease

There have been a number of studies published recently which extend what is known about the effects of vestibular dysfunction on brain volume, following the study by Brandt et al. (14) which demonstrated a bilateral atrophy of the hippocampus in patients with BVL. Dordevic et al. (15) studied 15 patients with chronic (at least 6 months), mild unilateral or bilateral vestibulopathy and compared them with healthy controls. None of the patients suffered from severe hearing loss as might occur in Meniere's Disease. They observed that the patients performed significantly worse on the clinical balance test, the triangle completion test for path integration (a test of spatial navigation) and the rotational memory test, but not on the Berlin intelligence structure test or the d2-R test for attention and concentration. However, in contrast to some previous studies [e.g., (14)], they found no significant differences in volumetric gray matter, including in the medial temporal lobe. Cohen et al. (16) studied patients with AD and cognitive impairment and found that they exhibited vestibular dysfunction according to Dix-Hallpike maneuvers and cervical vestibular-evoked myogenic potentials (cVEMPs), a measure of saccular function. However, they also found that patients with impaired cVEMPs exhibited significantly reduced left hippocampal volumes compared to those with normal cVEMPs. A number of previous studies have reported a link between abnormal otolithic function and AD [see (2) for a review]. Interestingly, there has been a recent report of punctate hippocampal lesions being associated with an acute vestibular syndrome (17). At the current time, there is no obvious explanation for the discrepancies relating to vestibular dysfunction and hippocampal atrophy, between the different studies.

Previous studies have reported that vestibular dysfunction increases the risk of AD by several-fold [see (2) for a review]. A number of studies have investigated the possibility of using aspects of vestibular function as potential biomarkers for AD. Wang et al. (18) compared visuo-spatial, executive and attentional function, and EEG and P300 responses, in 21 patients with age-related vestibular loss, 19 patients with cognitive impairment and 21 age- and sex-matched healthy controls. The three groups were also matched for hearing threshold and central auditory processing in order to exclude differences in hearing as a confounding factor. They found that the vestibular-impaired group exhibited deficits in these cognitive functions, a reduced P300 response and decreased gamma connectivity between the right Brodmann area 19 (B19, the right cuneus) and Brodmann area 7 (BA7) in the left superior parietal gyrus, in both the vestibular-impaired and cognitively-impaired group, relatve to the controls. The authors suggested that the changes in P300 and functional connectivity in the patients with age-related vestibular loss may serve as useful biomarkers for vestibular-related cognitive dysfunction. Ide et al. (19) have reported significantly poorer index of postural stability (IPS) scores in AD patients compared to those with mild cognitive impairment, in the closed eyes/hard surface condition. Biju et al. (20) have also reported that AD patients exhibit increased medio-lateral sway in both eyes open and eyes closed conditions.

Despite the evidence linking vestibular dysfunction and AD, there is no reason, based on currently published data, to think that there is a specific connection to this form of dementia, rather than dementia in general. For example, vestibular abnormalities have been detected in frontotemporal dementia, which also presents with an impairment of visuospatial function (21). It is possible that if vestibular loss does contribute to cognitive dysfunction, it is involved in many forms of dementia.

Putman et al. (22) have recently published the results of a study in which they showed that noisy galvanic vestibular stimulation (nGVS), which has been shown to enhance motor control in PD [e.g., (23)], enhanced task learning in a functional mobility task (navigating an obstacle course on a compliant surface with degraded visual cues) compared to sham controls. Interestingly, the benefits of the nGVS were maintained even following the cessation of the stimulus. However, the enhancement was not observed in a manual control task (using a joy-stick to null self-roll tilt in response to a pseudo-random disturbance in the dark).

### Animal studies

**Figure 3:**
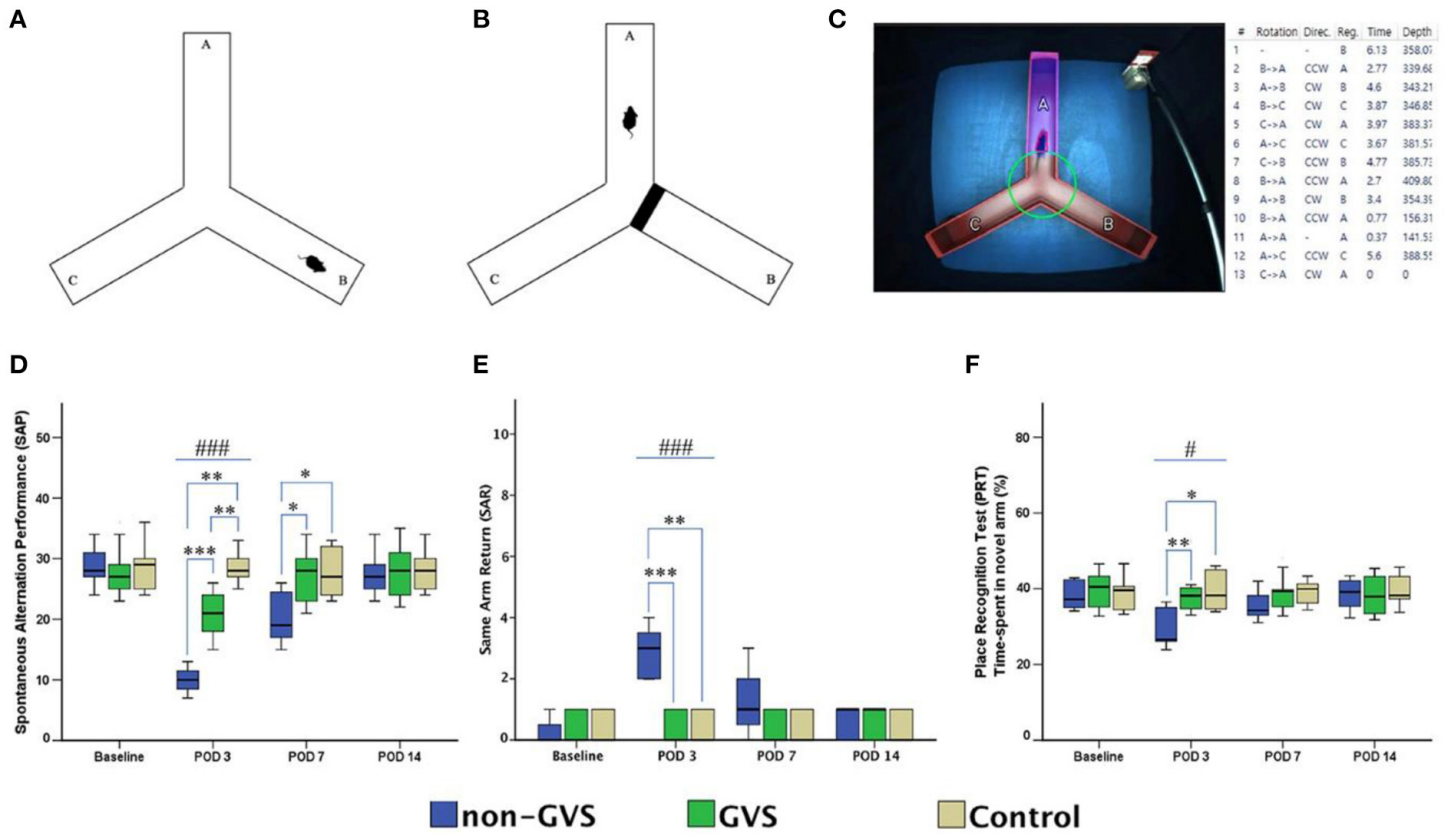
Evaluation of locomotor activities and spatial navigation through the Y maze test. The mice move freely within three arms **(A)**. Mice were trained with a block in the B arm for 3 min, then the block was removed, and the mouse activity for exploring the B arm was assessed, i.e., the place recognition test **(B)**. Mice activities in the three groups of non-GVS, GVS, and control groups were tracked and computed by analysis software in 6 min at four time points: baseline, and post-operative days (PODs) 3, 7, and 14 **(C)**. There was a significant difference between the groups in the spontaneous alternation performance at PODs 3 (χ^2^ = 17.11, *p* < 0.001, Kruskal–Wallis test). This decline in the non-GVS group continued until POD 7 as compared to the GVS group (*Z* = −2.12, *p* < 0.05) and control group (*Z* = −1.95, *p* < 0.05) **(D)**. There was also a significant difference between the groups in the same arm return at POD 3 (χ^2^ = 15.23, *p* < 0.001, Kruskal–Wallis test) **(E)**. The place recognition test indicates spatial reference memory and it shows a significant difference between the groups at POD 3 (χ^2^ = 7.63, *p* < 0.05, Kruskal–Wallis test) **(F)**. Values of significant difference were calculated by using the Kruskal–Wallis test for between groups and the Mann–Whitney *U-*tests for pairwise comparisons. *Significantly different between two groups; ^#^Significantly different between three groups; *^, #^*p* < 0.05; **^, *##*^*p* < 0.01; ***^, *###*^*p* < 0.001. Reproduced from Nguyen et al. (25).

**Figure 4:**
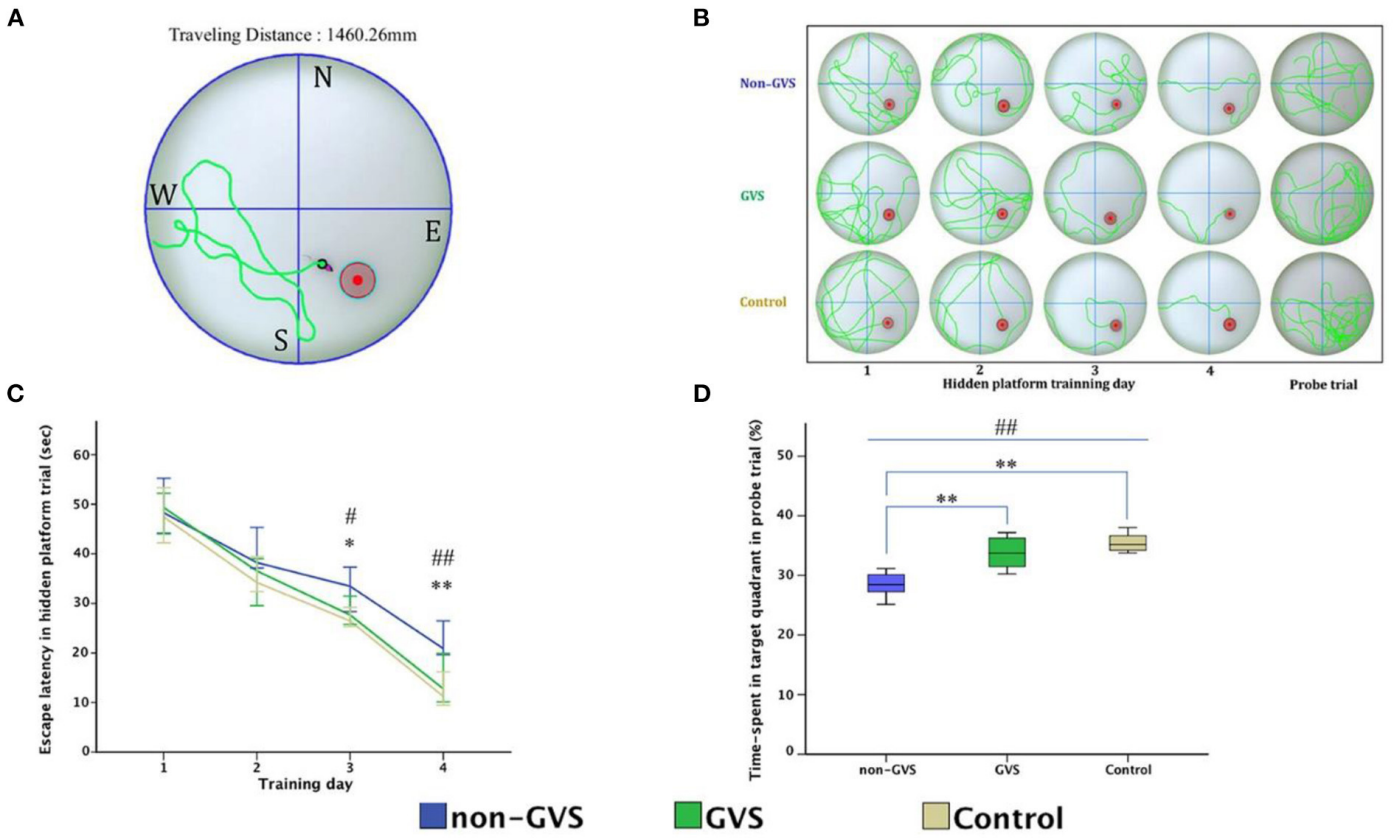
Evaluation of motor coordination and spatial navigation of mice through the Morris water maze (MWM). The analysis package divided the searching area into four quadrants, one of which contains the escape platform (red circle) **(A)**. The process of finding the escape platform from the starting point was tracked in the mice (pink) for 1 min **(A)**. Mice were trained with the visible platform at post-operative day (POD) 8 (not depicted) and hidden platform for 4 consecutive days (POD 10–13) and no platform in the probe trial at POD 14 **(B)**. Longer values of escape latency to find the hidden platform indicate an inadequate acquisition of spatial memory and navigation, which showed differences between groups at the last two training days (χ^2^ = 6.54, *p* < 0.05 and χ^2^ = 10.52, *p* < 0.01, Kruskal–Wallis test). Non-GVS mice had a longer escape latency (33.5 s on the third day and 20.9 s on the fourth day of hidden platform trials) than those of the GVS group (27.67 s, *Z* = −2.07, *p* < 0.05 on the third day and 17.39 s, *Z* = −2.73, *p* < 0.01 on the fourth day) and the control group (26.47 s, *Z* = −2.19, *p* < 0.05 on the third day and 11.25 s, *Z* = −2.61, *p* < 0.01 on the fourth day) (Mann–Whitney *U-*test) **(C)**. During the probe trial at POD 14, there was a significant decrease in the percentage of time spent in the target quadrant in the non-GVS mice [28.5% (26.2–30.6%)] compared to the control group [35.2% (34.0–37.3%), *Z* = −2.61, *p* < 0.01, Mann–Whitney *U-*test] **(D)**. GVS intervention substantially enhanced recovery of this deficit [33.7% (30.9–36.5%), *Z* = −2.73, *p* < 0.01, Mann–Whitney *U-*test], and they were no different from the control group **(D)**. *Significantly different between two groups; ^#^Significantly different between three groups; *^, #^*p* < 0.05; **^, *##*^*p* < 0.01. Values of significant difference were calculated by using the Kruskal–Wallis test for between groups and the Mann–Whitney *U-*tests for pairwise comparisons. Reproduced from Nguyen et al. (25).

Over the last 2 years, Nguyen et al. have published a series of studies which investigated the effects of GVS on spatial cognition in mice. In the first study, they compared animals with UVL that received GVS with UVL animals that did not, as well as a control group (24). Cognitive function was assessed at 3, 7, and 14 days using the Y maze and Morris Water Maze (MWM) tests. Importantly, the UVL was created using a surgical method so that it was more specific to the vestibular system than a chemical lesion. The GVS, which was a bipolar, sinusoidal current at 1 Hz, was subthreshold for inducing nystagmus and the cathodal electrode was positioned on the right (lesioned) side. GVS was delivered for 5 days post-UVL with 30 min sessions each day. Mice with UVL exhibited significant impairments in cognitive function, which were reduced by GVS treatment. This was the case for both the Y maze test and the MWM test (see Figures 3, 4). In a follow-up study, they compared left- and right-sided UVL and found that the spatial cognitive deficits differed depending on the side of the lesion (25). Spatial cognition was more impaired in the left-sided UVL group compared to the right-sided, suggesting a laterality effect, although GVS accelerated recovery in both groups when the cathode was on the side of the lesion (25).

**Figure 5:**
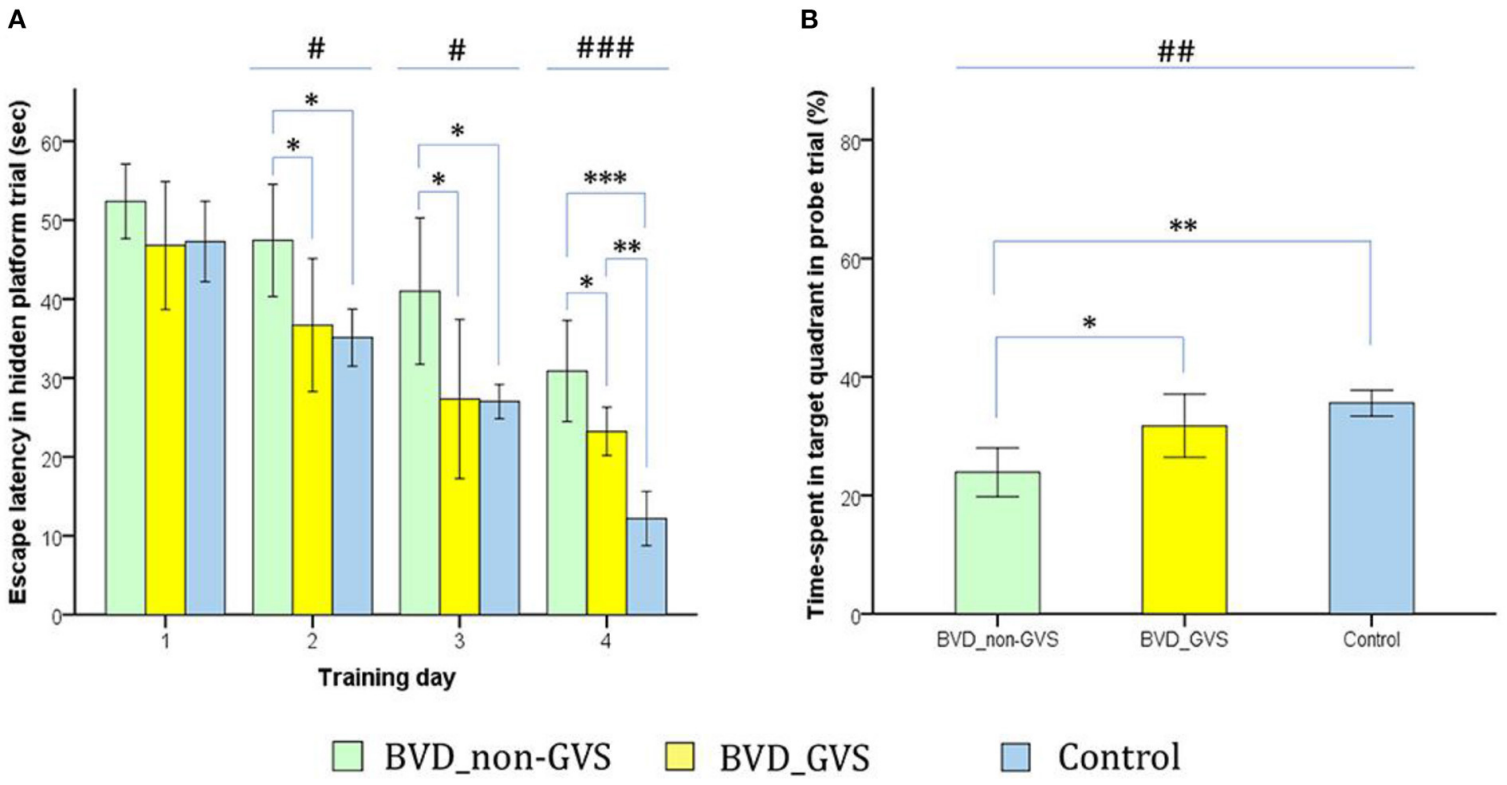
Evaluation of long-term spatial reference memory using the Morris water maze (MWM). The escape latencies to find the hidden platform gradually decreased through the training sessions, indicating ongoing learning. Longer values of escape latency to find the hidden platform indicate an inadequate acquisition of spatial memory and navigation. Differences between the groups were observed on training days (TDs) 2 (*p* = *0*.012, ANOVA), 3 (*p* = *0*.013, ANOVA), and 4 (*p* < *0*.001, ANOVA). The BVD non-GVS group had longer escape latency than the control group on TDs 2 (*p* = 0.022, Bonferroni test), 3 (*p* = 0.032, Bonferroni test), and 4 (*p* < 0.001, Bonferroni test). The BVD GVS group had shorter escape latency than the BVD non-GVS group on TDs 2 (*p* = 0.037, Bonferroni test), 3 (*p* = 0.028, Bonferroni test), and 4 (*p* = 0.024, Bonferroni test) **(A)**. Residual impairments in long-term spatial memory were also indicated by a lower percentage of time spent in the target quadrant (probe trial) on POD 14 in the BVD non-GVS compared with both the control (*p* = 0.001, Bonferroni test) and BVD GVS groups (*p* = 0.012, Bonferroni test) (*p* = 0.001, ANOVA) **(B)**. The values are indicated as the mean ± SD. Statistical significance was calculated using one-way ANOVA with *post-hoc* tests. *Significant differences between two groups; ^#^Significant differences among three groups: *^, #^*p* < 0.05; **^, *##*^*p* < 0.01; ***^, *###*^*p* < 0.001. Reproduced from Nguyen et al. (25).

In Nguyen et al. (26), they applied the same experimental paradigm to mice that had received an incomplete surgical BVL. Again, the effects of GVS were compared on performance in the Y maze and MWM at 3, 7, and 14 days post-op. Similar to previous studies [e.g., (27, 28)], they found that BVL impaired spatial memory in both the Y maze and MWM. However, GVS accelerated the recovery from the spatial memory deficits (Figure 5).

Taken together, the studies published since 2021 reinforce the previous evidence that normal vestibular function is necessary for intact cognition, especially spatial cognitive processes. More evidence for this has been published in both humans and animals; however, the human studies present a more complex and varied picture, with some studies showing less severe effects or more circumscribed effects [e.g., novel spatial information but not familiar spatial information (5)]. Inevitably, because the studies in patients involve an heterogeneous array of vestibular disorders, with different degrees of vestibular dysfunction, sometimes confounded by concurrent hearing loss, and different time courses, the effects of vestibular loss on cognition are bound to be more variable, and may be explained partly by Lacroix et al.'s (12) reference to Kahneman's Capacity Model of Attention. The evidence that vestibular loss increases the risk of AD is also gradually accumulating (2).

## The vestibular system and the hippocampus

Since the early 2000's, many animal studies have been published which show that the thalamus and hippocampus undergo abnormal plasticity following the loss of vestibular function (1). This has been followed by studies in patients with vestibular disorders which, in the majority of cases, have demonstrated structural changes in the hippocampus, such as bilateral atrophy [e.g., (14)]. A number of specialized spatially tuned neurons in the medial temporal lobe and thalamus are known to be important for spatial memory [see (29) for a review]. However, despite the fact that spatial memory deficits associated with vestibular loss are often attributed to the dysfunction of place cells in the hippocampus, there are still only two specific studies that have demonstrated abnormal place cell activity in the hippocampus (30, 31) and one study that has demonstrated abnormal grid cell activity in the entorhinal cortex (32). Although there have been no further studies of these neuronal types directly related to the effects of vestibular loss over the last 2 years, there have been some studies published which are relevant to understanding the effects of vestibular dysfunction on these areas of the brain.

Van Rompaey et al. (33) studied the effects of hearing and vestibular loss caused by the oral intake of allynitrile in mice, and found, similar to previous studies, deficits in spatial memory, using the Barnes Maze. They also used doublecortin immunohistochemistry to investigate the number of immature neurons in the hippocampus, as an indication of neurogenesis. Surprisingly, they found a significant decrease in doublecortin-+ve cells in the left, but not right hippocampus, compared to the control group. This result suggests that the combination of hearing and vestibular loss may impact on neurogenesis in the left hippocampus. Whether the immature neurons in these studies would have become place cells is, of course, unknown; however, a decrease in neurogenesis could have an impact on place cell numbers. Previous studies have reported an increase in cell proliferation following BVL (34), although GVS was found to reduce neurogenesis in the left hippocampus (35).

Historically, most hippocampal place cell studies have been conducted in freely moving rats and mice and there has been some question as to whether place cells behave similarly in primates. In the first study of spatial coding in freely moving macaques, Mao et al. (36) found hippocampal neurons that were more complex than those described in rodents and which were tuned to many different spatial variables, including horizontal position, head height, linear speed, azimuth head direction, head tilt, head-facing location in 3 dimensions, egocentric boundary (i.e., relative to the arena boundary) and angular head velocity. They observed that the firing of hippocampal neurons was mainly modulated by position (26%), speed (22%), and head direction (41%), with 26% exhibiting conjunctive firing. Interestingly, neuronal activity was strongly modulated by eye movement. This is very significant given the differences in eye movements between primates and rodents, i.e., eye movements in rodents are mainly reflexive, as opposed to voluntary saccadic and smooth pursuit movements. “Place cells”, as defined in rodents, were relatively rare but similar to other studies in monkeys. Only 1% of neurons showed grid-like modulation as in rodents. There was an intermittent, low frequency (~4 Hz) theta activity specific to movement onset, speed-dependent and to which many neurons were phase-locked. Theta phase precession was rare. This study strongly suggests that to understand the neural basis of spatial memory deficits associated with vestibular loss in the hippocampus, the differences in hippocampal processing of spatial information between rodents and primates is crucial to keep in mind. Keshavarzi et al. (37) have recently reported angular head velocity responses, highly dependent on vestibular input, in the retrospenial cortex of the mouse. Their gain was enhanced by the addition of visual information; one might speculate on how much more complex these cells could be in the primate retrospenial cortex.

**Figure 6:**
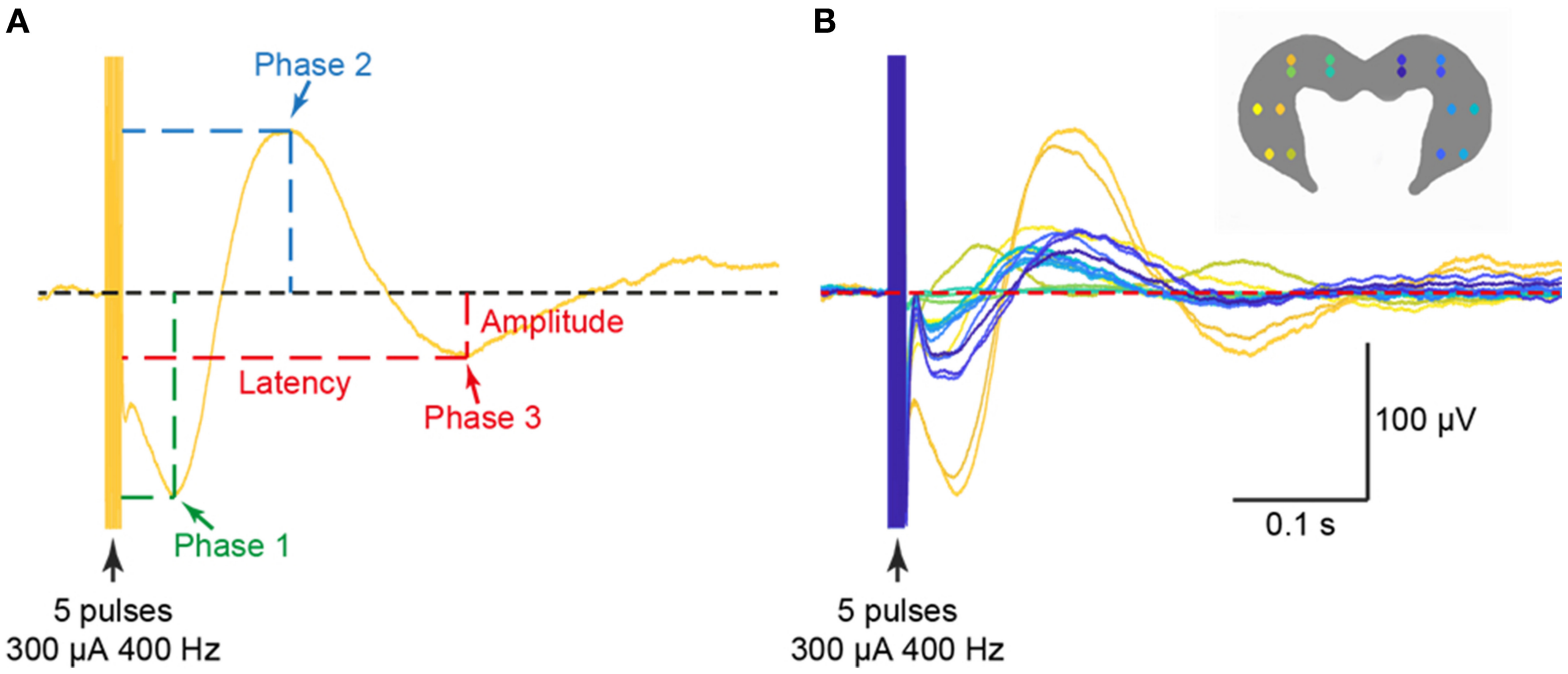
Examples of triphasic waveforms **(A)** from all 16 electrodes **(B)** evoked in the ipsilateral and contralateral hippocampus from electrical stimulation of the saccule. The stimulus used was 300 μA at 400 Hz. Reproduced with permission from Hitier et al. (38).

**Figure 7:**
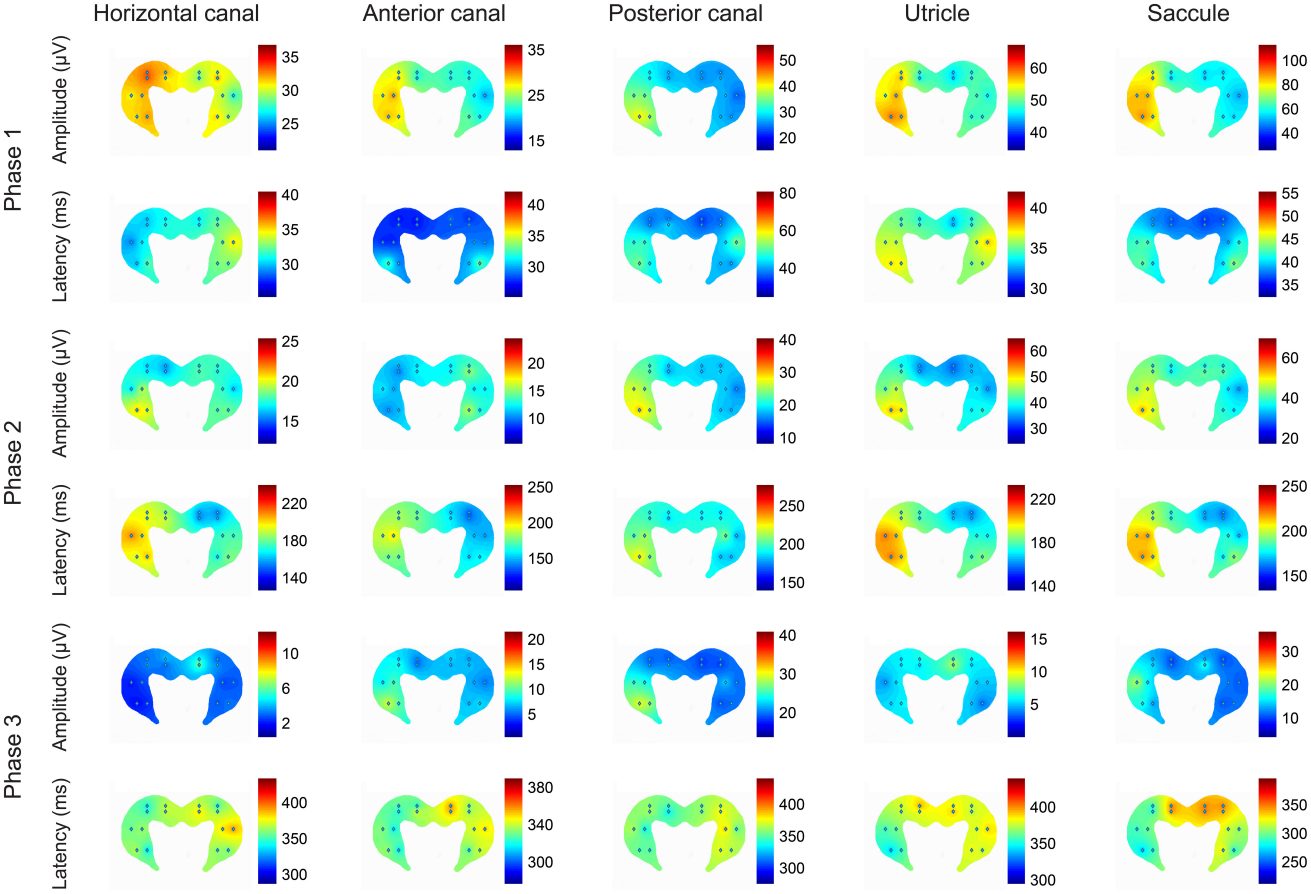
Heat plots of the patterns of amplitudes and latencies of the 3 phases of the local field potentials (LFPs) evoked in the hippocampus by electrical stimulation of the horizontal canal (HC), anterior canal (AC) or posterior canal (PC) ampullae, or the utricle or saccule, where the hot colors represent the highest amplitudes and the longest latencies. The heat maps represent the data from all of the rats used in the experiments. The left side on the figures represents the right hippocampus and is contralateral to the stimulation. This is a heatmap scaled by itself, i.e., every single heatmap has maximum and minimum colors. The advantage of this heatmap is that it helps to understand how stimulation of the different sensors activates different areas of the hippocampus. The stimulus used was 300 μA at 400 Hz in all cases. Reproduced with permission from Hitier et al. (38).

There has been recent progress in understanding the nature of the projections from the vestibular system to the hippocampus in the last 2 years. Hitier et al. (38) selectively electrically stimulated the different parts of the vestibular labyrinth in the rat—the horizontal, anterior, posterior semi-circular canals and the utricle and saccule—and recorded triphasic local field potential responses throughout the bilateral hippocampi using a 16 electrode microarray (Figure 6). They found responses of varying amplitudes and latencies throughout the dorsal and ventral hippocampus following stimulation of the different vestibular sensors, but with generally larger amplitudes on the side contralateral to the stimulation and in response to saccular and utricular stimulation (Figures 6, 7). The greater response to otolithic stimulation is consistent with the view that information about gravitational vertical is especially important for the hippocampus [see (39) for a review]. The responses were polysynaptic and long latency in all cases, and it remains to be seen how much and what kinds of vestibular input are transmitted to the hippocampus *via* various pathways from the vestibular nucleus vs. the cerebellum.

Taken together, the studies of vestibular-hippocampal interaction published over the last 2 years, although there have been only a few, have increased our understanding of the way the hippocampus may process vestibular information. Up to now, there has been a wealth of evidence demonstrating that the hippocampus becomes abnormal following vestibular loss, but less information about *how* the hippocampus uses vestibular sensory input. The study by Van Rompaey et al. (33) suggests that vestibular input may actually be necessary for the normal level of neurogenesis in the hippocampus. The study by Hitier et al. (38) shows that vestibular input to the hippocampus is much more widespread than previously thought and also may be lateralized, even in rats. Perhaps the most important study is that by Mao et al. (36), which demonstrates for the first time that the nature of spatially-responsive hippocampal neurons in primates may be much more complex than predicted from studies in rats and mice and that therefore the effects of vestibular loss may be similarly complex.

## The vestibular system and the striatum

Studies of connections between the vestibular system and the basal ganglia date back to the 1960's, with speculation about how vestibular information might contribute to the control of non-reflexive movement [see (3) for a review]. However, the results of early electrophysiological studies have been inconsistent and difficult to reconcile; therefore vestibulo-strital pathways have remained a mystery [see (3) for a review]. Connections between the vestibular system and the striatum have often been investigated because of their possible relevance to PD. However, the dorsal striatum (i.e., the putamen and the caudate nucleus) is also known to be important in learning and memory and interacts with the hippocampus to compare cognitive information [see (40) for a review].

**Figure 8:**
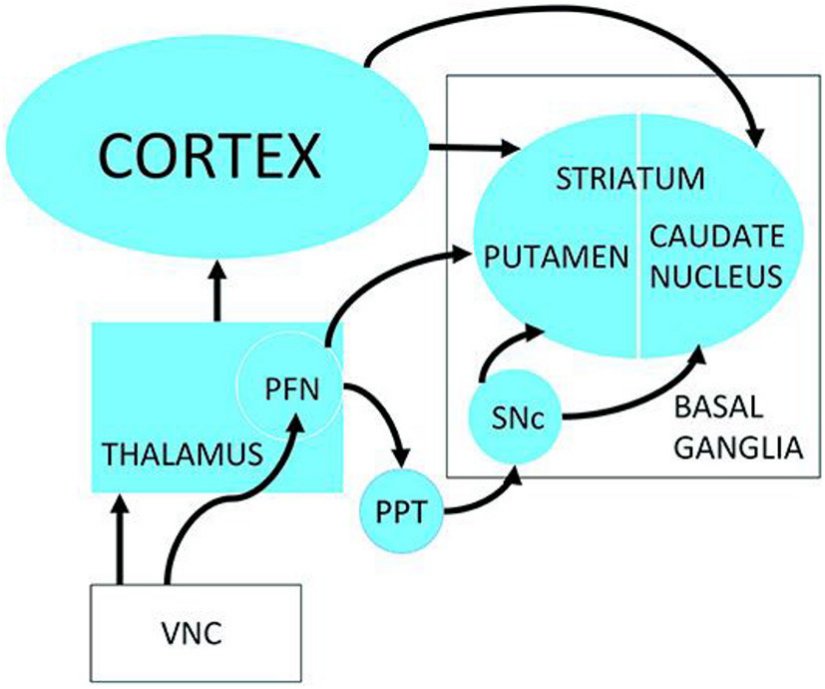
Possible neuronal pathways connecting the vestibular nucleus complex to the striatum. PFN, Parafascicular nucleus; PPT, pedunculopontine tegmental nucleus; SNc, Substantia nigra pars compacta; VNC, vestibular nucleus complex. Reproduced from Smith (3).

Since 2021 there have been a number of major studies relating to vestibulo-striatal interactions and PD. One of the possible pathways for the transmission of vestibular information to the striatum is *via* the pedunculopontine tegmental nucleus (Figure 8), which, along with the striatum, has been demonstrated to undergo plasticity following BVL [(41–43); see (44) for a review]. Özkan et al. (45) have recently reported the results of a study in which they injected fluoro-gold tracer into the pedunculopontine tegmental nucleus of rats and observed labeling in the vestibular nucleus both ipsilateral and contralateral to the injection. Similar results were obtained in humans using diffuser tensor imaging and data from the Human Connectome Project.

Bohnen et al. (46) studied vesicular acetylcholine transporter expression using positron emission tomography imaging in PD patients, as an indicator of the integrity of cholinergic pathways. They found evidence of cholinergic deficits in many different brain regions but one of them was the cholinergic pathway arising from the medial vestibular nucleus. In a further study, the authors demonstrated that such cholinergic deficits were specifically associated with postural instability and gait difficulties (47).

Antons et al. (48) undertook a large longitudinal dual tracer study in rats following BVL, using positron emission tomography with computer tomography to measure functional and structural plasticity throughout the brain at 1, 3, 5, 7, and 9 weeks post-lesion. They found significant decreases in glucose metabolism in both the left and right striatum, coupled with increases in synaptic density. The BVL was induced by the intratympanic injection of bupivacaine and p-arsanilic acid; therefore, effects on the auditory system could not be excluded. However, these results suggest that the striatum undergoes substantial plasticity following BVL, consistent with the results of previous studies [e.g., (41)].

Hawkins et al. have undertaken a series of systematic studies of vestibulo-ocular reflex and otolith function in a sample of 40 patients with PD and 40 healthy controls. Using the video head impulse test, they found, somewhat surprisingly, no significant differences in the gain of horizontal or vertical vestibulo-ocular reflex function (49). This is in contrast to some previous studies [see (3) for a review]. They obtained similar results using the suppression head impulse paradigm, although they did observe saccadic dysfunction in PD patients (50). In studying otolith function, they found that the PD patients exhibited significantly more absent cervical-evoked myogenic potentials to both clicks and taps, indicating saccular dysfunction, consistent with previous studies [(51); see (3) for a review]. In a further study (52), they used a virtual reality task and demonstrated that PD patients displayed poorer balance which correlated with the severity of the disease, age, vestibulo-ocular reflex function and proprioceptive ability. These studies are particularly significant because of the size of the sample of PD patients and also the use of the same number of controls.

As with AD, although PD has been the focus of attention for the effects of vestibular loss on the basal ganglia, there is no reason to think that this is specific to PD, and it is conceivable that vestibular loss is associated with other basal ganglia disorders. For example, neuronal loss in the vestibular nuclei has been reported in Huntington's Disease [e.g., (53)].

In recent years GVS has been investigated extensively as a potential treatmenf for PD [see (54) for a review]. In what is the most recent study, Lee et al. (23) investigated 18 patients with PD and 20 healthy controls using a simple reaction time task while receiving subthreshold GVS. Because there has been considerable controversy surrounding the type of GVS that might have optimal effects, they decided to compare 9 different types of GVS, including random noise GVS, which has been used most often, and 7 different kinds of multisine stimuli, in both on- and off-medication conditions. Since random noise GVS is supposed to work by the stochastic resonance principle, the purpose of comparing sine wave stimuli of different frequencies (4–200 Hz) to random noise GVS was to test whether stochastic resonance was necessary to improve PD symptoms and also to determine whether the sine wave GVS could be optimized to individuals. They found that the multisine-γ GVS (30–50 Hz) was associated with the shorter response time in both the PD off-medication and control groups compared to the random noise group. The response time for the PD off-medication groups also decreased during the multisine-β (13–30 Hz) GVS. The authors concluded that the optimal GVS frequency to ameliorate PD symptoms may vary considerably between patients and that random noise GVS is not necessarily the best GVS stimulus.

Taken together, the few studies of vestibulo-striatal interaction and vestibular modulation of PD have advanced our understanding of this area. Although the details are still to be elucidated, it is becoming clear that the pedunculopontine tegmental nucleus, which is known to project to the striatum, is also connected to the vestibular nucleus; furthermore, it is evident that PD is associated with major changes in cholinergic pathways involving the vestibular system. The study by Antons et al. (48) reinforces previous evidence that vestibular loss is associated with major functional changes in the striatum. Evidence continues to be reported that GVS can modulate the symptoms of PD (23). Finally, a series of recent studies by Hawkins et al. (49–52) has suggested that vestibular symptomatology in PD may be most prominent for the otoliths and not for the semi-circular canals and vestibulo-ocular reflexes.

## Conclusions

Many studies have been published during the last 2 years which have had an impact on our understanding of the contribution of vestibular function to cognition, the hippocampus and the striatum. A number of these studies have reinforced and extended our understanding of the cognitive effects of vestibular dysfunction (4–8, 10). New evidence has been reported on the possible association between vestibular dysfunction and AD (16), as well as vestibular deficits as possible biomarkers for the disease (18–20). Although Dordevic et al. (15) reported no significant change in hippocampal volume associated with vestibular loss, Cohen et al. (16) found that in AD patients, cervical-evoked myogenic potential deficits were associated with a reduction in the volume of the left hippocampus. Further evidence has been published in support of the use of GVS to enhance recovery from vestibular loss in rats (24–26). Consistent with previous studies, Hitier et al. (38) have demonstrated that vestibular input is extensively represented in the rat hippocampus and that all of the vestibular sensors—the horizontal, anterior and posterior semi-circular canals and the utricle and saccule—transmit information there in a stratified fashion, with preferential input from the otoliths and to the contralateral side.

In terms of the striatum, recent studies indicate that major changes occur in the cholinergic pathways involving the vestibular nucleus in PD (46, 47), and that BVL is associated with plasticity in the bilateral striatum. Hawkins et al. (49–52) have also published a series of systematic, well controlled studies of PD patients showing that vestibulo-ocular reflex function is not significantly affected but that cervical-evoked myogenic potential function is degraded.

Taken together, these studies support the idea that vestibular information is important for normal hippocampal and striatal function, may be relevant to AD and PD, as well as other disorders, and that a greater understanding of the processing of vestibular input in these two structures, and how they interact, may benefit our understanding of neurological disorders.

## Author contributions

The author confirms being the sole contributor of this work and has approved it for publication.

## Funding

This research was supported by a Grant from the Health Research Council (HRC) of New Zealand 20/399.

## Conflict of interest

The author declares that the research was conducted in the absence of any commercial or financial relationships that could be construed as a potential conflict of interest.

## Publisher's note

All claims expressed in this article are solely those of the authors and do not necessarily represent those of their affiliated organizations, or those of the publisher, the editors and the reviewers. Any product that may be evaluated in this article, or claim that may be made by its manufacturer, is not guaranteed or endorsed by the publisher.
